# Sequence analysis reveals asymptomatic infection with *Mycoplasma hominis* and *Ureaplasma urealyticum* possibly leads to infertility in females: A cross-sectional study

**DOI:** 10.18502/ijrm.v19i11.9910

**Published:** 2021-12-13

**Authors:** Chinyere Charity Ezeanya-Bakpa, Nneka Regina Agbakoba, Charolette Blanche Oguejiofor, Ifeoma Bessie Enweani-Nwokelo

**Affiliations:** ^1^Department of Medical Laboratory Science, Nnamdi Azikiwe University, Awka, Nigeria.; ^2^Department of Microbiology and Biotechnology, Caleb University Lagos, Nigeria.; ^3^Department of Obstetrics and Gynaecology, Nnamdi Azikiwe University Teaching Hospital, Nnewi, Nigeria.

**Keywords:** Mycoplasma hominis, Ureaplasma urealyticum, Genetic linkage, Asymptomatic infections, Infertility.

## Abstract

**Background:**

Genetic evidence of asymptomatic *Mycoplasma hominis* (*M. hominis*) and *Ureaplasma urealyticum* (*U. urealyticum*) infection associated with infertility among females is lacking because suitable high throughput molecular methods have not been applied.

**Objective:**

This study aimed to explore the occurrence of *M. hominis* and *U. urealyticum* in the genital tract of females with asymptomatic infection and infertility as well as determine their genetic relatedness.

**Materials and Methods:**

The study group included 100 asymptomatic females and 31 females diagnosed with infertility. Sequencing of the 16S rRNA gene following DNA extraction was performed directly from endo-cervical swabs. Phylogenetic analysis established the genetic linkage between the isolates from both groups.

**Results:**

In asymptomatic females, *M. hominis* and *U. urealyticum* were detected with a prevalence of 8% and 2% respectively. Among females with infertility, the prevalence was 6.45% and 3.23% for* M. hominis* and *U. urealyticum *respectively. In both groups, *M. hominis* occurred significantly more frequently. Phylogenetic analysis revealed three distinct clusters in both groups: two with already characterized *M. hominis* and *Ureaplasma *species (28.6% of the overall *Mycoplasma* spp.) and one distinct cluster matched with *U. urealyticum*. Furthermore, all *M. hominis* from asymptomatic females clustered significantly with infertility contrary to *U. urealyticum*. The *M. hominis* cluster was significantly linked to two strains from China.

**Conclusion:**

The sequence analysis of *Mycoplasma* and *Ureaplasma* in the genital tract of asymptomatic and infertile females showed significant association; therefore, it is paramount to consider them as possible etiologic agents of infertility and genital infection, especially when the etiology of infertility is unknown.

## 1. Introduction

*Mycoplasma hominis* (*M. hominis*) and *Ureaplasma urealyticum* (*U. urealyticum*) are members of the family *Mycoplasmataceae* which also contains *U. parvum*. They are distinctive for their remarkably small size when compared to other bacteria of public health significance. *M. hominis* and *U. urealyticum* are considered among the most prevalent genital *Mycoplasma* (GM) in Nigeria, with a significant global distribution as well (1). These bacteria thrive in the genital tract of a significant number of apparently healthy females (2); however, the infections caused may demonstrate the classical manifestation of clinical symptoms. The genital colonization of *M. hominis*, *U. parvum* and *U. urealyticum* have been reported to vary based on age. Females of reproductive age have been reported as the most susceptible, with a prevalence of 3.5-56.4% (3-5).

Infertility is a serious public health concern globally, affecting an estimated 22 million women of which 56% are within the ages of 15-45 yr (6). According to the World Health Organization's epidemiologic definition, the term “infertility” designates the failure of conception after regular attempts for pregnancy by `women of reproductive age' over a time period of ≥ two yr (7). There is an increasing tide of infertility in Sub-Saharan African countries (8-10) and Nigeria is not an exception, and the majority of infertility in females is of inexplicable origins.

*M. hominis* and *U. urealyticum* contribute significantly to asymptomatic genital infections in females (11). They have established associations with bacterial vaginosis (12, 13), cervicitis, and the former has a strong association with pelvic inflammatory disease (14). However, there has been a difference of opinion in the involvement of *M. hominis* in infertility. The cervix of sexually active females is readily colonized by the class *Mollicutes* (which includes *M.* *hominis* and *U. urealyticum and U. parvum*) which promotes their pathogenicity (15).

There has been a rising prevalence of *M. hominis* and *U. urealyticum* in females with infertility. It has been reported that *M. hominis* and *U. urealyticum* can be found in an estimated 2.0-40.5% of females with infertility globally (16, 17). Similarly, the prevalence among asymptomatic females has been reported to range from 6.9 to 16.1% (18, 19). Without appropriate prevention, diagnosis, and treatment of *M. hominis* and *U. urealyticum*, infections among asymptomatic females can generate severe disease sequelae such as infertility (20). We hypothesized that *M. hominis* and *U. urealyticum* present in the reproductive tract of asymptomatic females undetected can result in infertility with unknown etiology.

The present study aimed to establish the genetic relatedness of *M. hominis* and *U. urealyticum* isolated from both asymptomatic females and females with infertility in Nigeria.

## 2. Materials and Methods

### Subjects

This cross-sectional study included 100 healthy females, aged 15-49 yr (mean age 30.5 ± 5.89 yr) and 31 females diagnosed with infertility of a similar age group (15-45 yr), within a 12-month period from March 2017 to February 2018 who were attendees of the Obstetrics and Gynaecology Clinic of Central Hospital Benin City, Nigeria. Other inclusion criteria were sexually active females and females not on antibiotic therapy within the last three months prior to the time of the study. The exclusion criteria were females diagnosed with a symptomatic genital infection and females who had been administered or were on treatment with antibiotics within the six months prior to the time of the study. A self-reported standardized questionnaire was used to obtain socio-demographic variables from all of the females. Sample size was determined at 95% confidence interval using 0.05 degree accuracy with a prevalence of 8.5% reported among females aged 15-45 yr in South Africa (21).

### Sample collection and analysis

An endo-cervical swab sample was collected from each subject targeted for *Mycoplasma* and *Ureaplasma* detection. The endo-cervical swab was sluiced in 0.4 ml of sterile phosphate buffered saline (Oxoid, United Kingdom) for 30 sec and then was immediately transported at 4°C. The supernatant was used for DNA extraction and afterward *Mycoplasma *and* Ureaplasma *species were detected via the molecular approach. Genomic DNA was extracted from the supernatant of the centrifuged inoculated phosphate buffered saline using the commercial extraction reagent kit “QiaQuick DNA kit” (Zymo Research, Germany) in compliance with the manufacturer's instructions, and was subsequently stored at -20°C. The concentration of the purified DNA was measured by spectrophotometry (Nano Drop 2000 Spectrophotometer, Thermo Scientific, Wilmington, United States). The commercial master mix with standard buffer “OneTaq Quick load 2X” (New England Bio Labs, United Kingdom) was included for the polymerase chain reaction (PCR) amplification. The final mix of 25 µl contained 1 µl of 25 µM of each primer, 12.5 µl of the master mix, 5 µl of genomic DNA template and 5.5 µl of sterile nuclease-free water (Amresco, United Kingdom). The negative and positive controls included: no DNA template and genomic DNA of GM (size 280 bp) respectively. Genus-specific primers for the 16S rRNA gene of GM (GPO and MGSO) were designed and supplied by Inqaba Biotechnology, Pretoria, South Africa according to previously reported design (22). Primer sequences were confirmed to have high specificity for the template sequence by checking for similarity to other known sequences with Blast (www.ncbi.nlm.nih.gov/blast/Blast.cgi). A 10 µl of the original PCR sample was analyzed by conventional 1% agarose gel electrophoresis. The DNA was stained with ethidium bromide and photographed with an automated trans-illuminator. Amplicon products of 280 bp were considered positive.

### Sequence and phylogenetic analysis of *Mycoplasma* and *Ureaplasma*

The capillary Sanger/dideoxy method was employed for the sequencing of the PCR products in both directions (forward and reverse) (Inqaba Biotechnology, Pretoria, South Africa), following the purification of the PCR amplicon with Sephadex powder and the quality control test. Strains were then distinguished as *M. hominis*, *U. urealyticum* and *U. parvum* using 16S rRNA sequencing. Sequences were submitted to the National Centre of Biotechnology Information. Species classification was accomplished through the phylogenetic analysis of the GM sequences and likewise, genetic relatedness of the isolates. Furthermore, 16S rRNA referenced sequences for both genital *Mycoplasma* and *Ureaplasma* were downloaded (available at the GenBank database). Genetic and phylogenetic analyses were done on the MEGA software (MEGA 7.0 version), and sequences were also aligned using the CLUSTALW algorithm (23).

### Ethical considerations

Ethical approval for the study was issued by the State Hospitals' Management Board Research Ethics Committee according to the Declaration of Helsinki (Code: A732/T/33). A signed consent form was obtained from all participants included in the study.

### Statistical analysis

The phylogenetic tree was generated using the maximum likelihood method and its dependability was established via bootstrapping analysis of 1,000 replicates. A cluster with ≥ 70% of the permuted trees was considered as a significant association if it was present. The number/percentage was used as descriptive statistics for the obtained data.

## 3. Results

Our study was a cross-sectional study involving 31 females with unexplained infertility out of which 45.16% and 54.84% had primary and secondary infertility respectively. Most of the subjects were married (74.19%) and had had an abortion (67.74%) (Table I). In addition, 100 asymptomatic (healthy) females of reproductive age (15-49 yr) were also recruited. The overall prevalence of genital *Mycoplasma*/*Ureaplasma *for both groups was 14/131 (10.7%); more specifically, the prevalence in asymptomatic females was 11/100 (11.0%) and in infertile females it was 3/31 (9.7%). The sequence analysis of the endo-cervical swabs confirmed the identification of the species among the subjects. The sequence analysis of the 14 *Mycoplasma* positive PCR products isolated from the endo-cervical swabs revealed eight cases of *M. hominis* in the asymptomatic subjects (8.00%), two of *U. urealyticum* (2.00%), and one of *U. parvum* (1.00%), and two cases of *M. hominis* in the infertile subjects (6.45%), one of *U. urealyticum* (3.23%), and none of *U. parvum* (0%).

To infer the evolutionary relationship amongst the genital *M. hominis* and *Ureaplasma *species, a phylogenetic tree based on their genetic sequences was obtained by the maximum likelihood method, classifying the *Mycoplasma* and *Ureaplasma *species from both groups as well as the genetic relatedness between various isolates of the same species. 19 sequences of *Mycoplasma* and *Ureaplasma* were aligned just before constructing the phylogenetic tree. 14 strains' sequences (submitted to the GenBank with the following assigned accession numbers: MG279049, MG279048, MG279050, MG279051, MG279052, MG279054, MG279055, MG279056, MG279057, MG281959, MG281961, MG388338, MG388339, MG388340) were inferred with four *M. hominis* reference strains (accession numbers AJ002265, NR041881, EU596508 and EU596509) and one *U. urealyticum* reference strain (accession number: AF073448). The percentage of sequence similarity covered by reads in this study was 99.5%.

**Figure 1:**
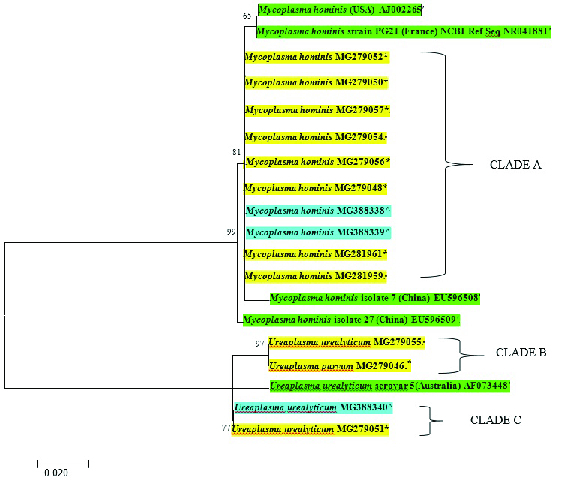
Maximum likelihood tree of genital *Mycoplasma* isolates including all identified and reference sequences. The isolated organism from the asymptomatic subjects, females with infertility and reference strains were highlighted in yellow*, blue ∧ and green ' respectively.

In figure 1, the bootstrap/confidence values (CV) are indicated on all branches. Here, species clustered into three significant clusters. Clustering was indicated by bootstrapping (> 70% of permuted trees). The strains of the present study were designated with the name of the organism and their respective accession numbers. All *M. hominis* strains from both groups (asymptomatic and infertile subjects) were found to be of a common clade (Clade A) with significant phylogenetic linkage (significant association) with CV of 81% and 99% to two Chinese strains: EU596508 and EU596509 respectively (although these were not of a common clade). Similarly, *U. urealyticum* MG279051 (asymptomatic-associated) had a significant linkage (CV = 77%) with the infertility-associated *U. urealyticum* MG388340, which was also found to have a common clade (Clade C). Furthermore, *U. urealyticum* and *U. parvum* (both from asymptomatic subjects) had a common clade (Clade B).

**Table 1:** Socio-demographics of the females in the study


**Variables**	**Females with infertility**	**Asymptomatic females**
** Age group (yr)**		
**15-19**	1 (3.23)	3 (3.00)
**20-24**	3 (9.68)	23 (23.00)
**25-29**	7 (22.58)	23 (23.00)
**30-34**	7 (22.58)	18 (18.00)
**35-39**	8 (25.80)	17 (17.00)
**40-44**	4 (12.90)	8 (8.00)
**45-49**	1 (3.23)	8 (8.00)
** Marital status**		
**Married**	23 (74.19)	37 (37.00)
**Single**	8 (25.81)	63 (63.00)
** Education**		
**Primary**	5 (16.13)	56 (56.00)
**Secondary**	10 (32.26)	38 (38.00)
**Tertiary**	16 (51.61)	6 (6.00)
** Type of infertility (unexplained)**		
**Primary**	14 (45.16)	Not applicable
**Secondary**	17 (54.84)	Not applicable
**Abortion**	
**Yes**	21 (67.74)	53 (53.00)
**No**	10 (32.26)	47 (47.00)
Data presented as n (%)		

## 4. Discussion

According to the nucleic amplification assay test results of our study, 10.7% of cervical swabs contained GM, indicating that these organisms are part of the cervical microbiome. The standard microbiological method of conventional PCR was seemingly appropriate for the detection of *M. hominis* and *U. urealyticum*; the existence of other species such as *U. parvum* in the cervix was not left undetected.

Asymptomatic females are rarely screened for GM. However, GM should be considered, especially when females are sexually active. The present study among seemingly healthy sexually active females in Nigeria demonstrated that 11.0% of these asymptomatic females were infected with GM. *U. urealyticum* was reported in 14.3% of the asymptomatic subjects and these are potential future patients. The prevalence of *U. urealyticum* has been previously reported among asymptomatic (healthy) females from Norway and Portugal as 8.6% and 28.4%, respectively (24, 25).

The occurrence of *M. hominis* among the asymptomatic subjects (8.0%) in our study does partially corroborate the findings reported from sexually active Portuguese females of reproductive age (8.5%) (25). Both studies used similar detection methods but different sample collection methods.

Previous studies have shown that the female genital tract of sexually active females tolerates and supports the growth of pathogenic mycoplasmas (26). *M. hominis, U. urealyticum* and *U. parvum* are exclusively human pathogens, largely spread through sexual contact; thus, all of the mycoplasmas and ureaplasmas isolated in this present study are likely of human origin, acquired via the transmission chain of genital tract infections.

There is a rising tide of unexplained infertility in females who are seldom tested for *M. hominis* and *Ureaplasma *spp. Given the reported occurrence of GM (9.7%) in our study, infertility patients should be considered as a high-risk group for *M. hominis* and *U. urealyticum*. This is in accordance with the study of Sleha and colleagues (17) who reported detecting *M. hominis* and *U. urealyticum* in 88 infertile women. Their results are comparable to ours, though *M. hominis* had a higher recovery rate in our study. The difference in sample size could explain the variation in these results despite the use of a similar sample type (endo-cervical swabs). Regarding species, 6.45% and 3.23% of women with infertility were positive for *M. hominis* and *U. urealyticum* respectively. A study by Min and co-workers (27) similarly reported that *M. hominis* can be an infectious cause of female infertility. A better understanding of the disease sequelae is needed for determining the best treatment regime and control measures as *M. hominis* and *Ureaplasma *spp can persist for months or even years in infected individuals.

There are limited data regarding the genetic relatedness among *M. hominis* and *Ureaplasma *spp isolated from asymptomatic and infertile females. The novelty of this contemporary study is the confirmation of the genetic relatedness of *M. hominis* (with a controversial role in female infertility) isolated from both asymptomatic and infertile subjects. All *M. hominis* strains from both groups were found to be of a common ancestor and had significant linkage to two Chinese strains, unlike the *U. urealyticum* strains. This finding corroborates the discriminate spread of sexually transmissible *M. hominis* and *Ureaplasma *spp irrespective of presentation of symptoms. This is supported by Gu et al. (28).

Here, we have been able to explicitly elucidate the genetic relatedness of *M. hominis* and *U. urealyticum* strains from both groups (asymptomatic and infertile subjects) with 81% and 77% confidence values respectively, which are indicative of significant linkage. Both the asymptomatic-associated species and infertility-associated species were clustered in common clades (Clade A and Clade C). This finding is inconsistent with a study by Boujemaa and co-authors (29), which described Tunisian clinical isolates of gynecological infection-associated and infertility-associated *M. hominis* clustering into different lineages. Our data indicated that *M. hominis* and *U. urealyticum* can result in serious sequelae (infertility) when left undetected and untreated. To the best of our knowledge, this is the first study to establish a genetic relatedness among *M. hominis* and *U. urealyticum* isolated from the cervix of asymptomatic and infertile females in Nigeria and to institute the disease sequelae associated with *M. hominis* and *U. urealyticum* among females.

A substantial number of research studies have concentrated on the implications of *Ureaplasma* spp. bacteria in pregnancy, infertility and other gynecological complications (3). The dearth of genomic reports regarding the genetic relatedness of ureaplasmas, ascertaining their involvement in infertility, sets a drawback in understanding the possible association between infertility and ureaplasmas. Interestingly, our study reports for the first time the presence of genetic relationships among ureaplasmas isolated from both infertile and asymptomatic sexually active females.

### Limitations of the study

The key limitation of our study was the relatively small sample size; accordingly it was a preliminary study. Studies with a larger sample size are recommended for further analyses or confirmation of the results.

## 5. Conclusion

The sequence analysis of *M. hominis* and *U. urealyticum* in the genital tract of females with infertility and asymptomatic infection showed significant associations; therefore, it is paramount to consider them as possible etiologic agents of infertility and genital infection regardless of the manifestation of clinical symptoms, especially when the etiology of infertility is unknown.

##  Funding information

There is no funding information to disclose for this study.

##  Conflict of interest

The authors declare no conflict of interest of any kind.
